# Supplementary material for: Complexome profiling on the Chlamydomonas lpa2 mutant reveals insights into PSII biogenesis and new PSII associated proteins
Source: J Exp Bot. 2021 Aug 26;73(1):245–62. doi: 10.1093/jxb/erab390 (PMC8730698; doi:10.1093/jxb/erab390)
Supplement: erab390_suppl_Supplementary_Dataset_S1 [file erab390_suppl_supplementary_dataset_s1.zip › Supplemental Dataset 1 - Excel List and all profiles/plots/DLE2_Cre11.g468450.html]

Trivial name: DLE2  
  
Euclidean distance: 14568.12  
Mean Intensity (WT): 295.79  
Mean Intensity (Mut): 733.14  
Distance: 19.87  
  
MapMan: cell.motility.eukaryotes.axonemal dyneins.inner arm.monomeric species;cell.motility.eukaryotes.deflagellation;cell.motility.eukaryotes.basal bodies;cell.organisation;DNA.synthesis/chromatin structure  
  
p value of intensity sums Welch test: 0.0935
